# Supplementary figures and images for: The Effect of Exogenous Melatonin on Eating Habits of Female Night Workers with Excessive Weight
Source: Nutrients. 2022 Aug 19;14(16):3420. doi: 10.3390/nu14163420 (PMC9412377; doi:10.3390/nu14163420)

Figure S1. Study flow chart.

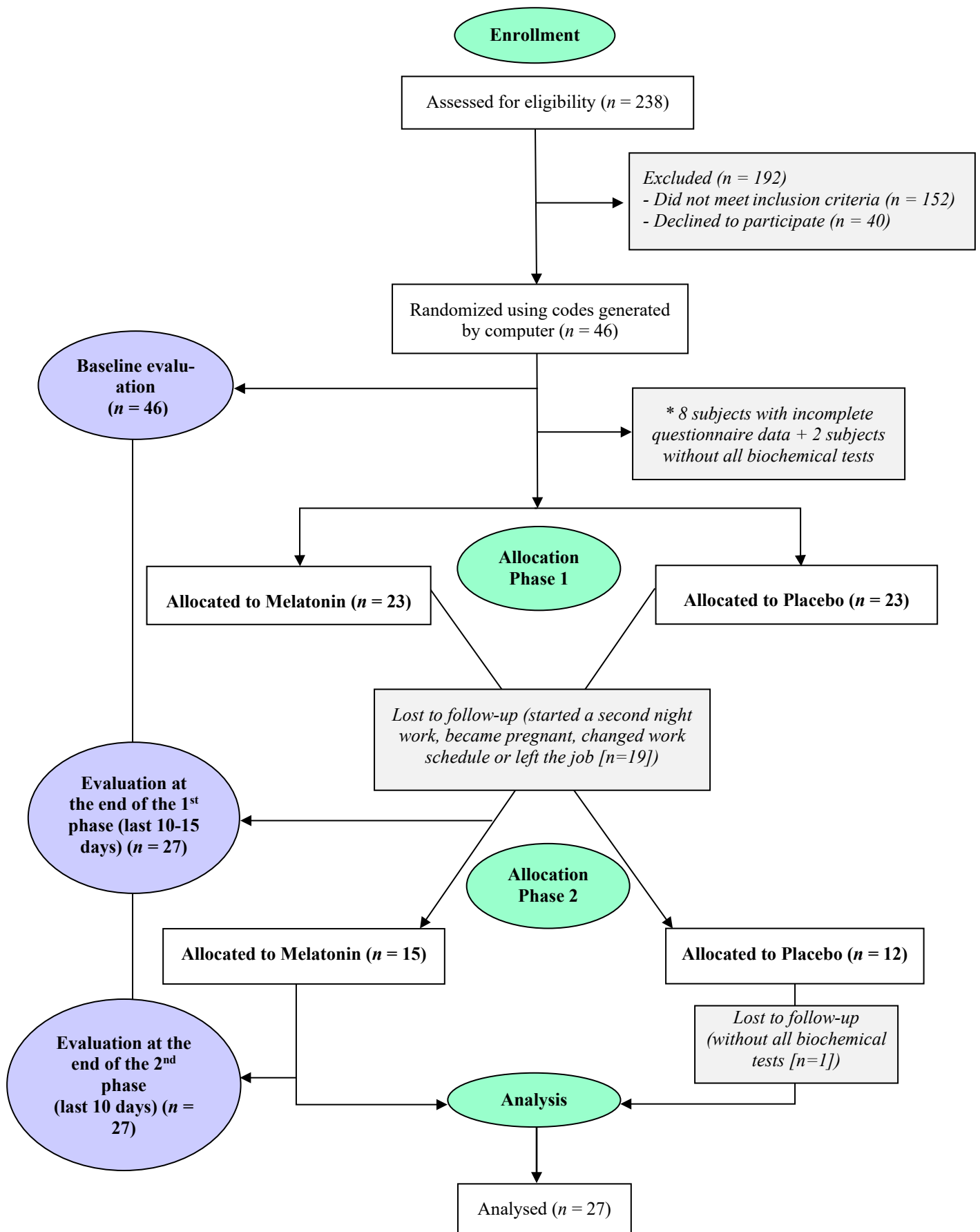

Supplement: Supplementary file 1 [file nutrients-14-03420-s001.zip › nutrients-1850676-supplementary.pdf]
